# Editorial: Molecular mechanisms and therapeutic strategies in inflammation

**DOI:** 10.3389/fimmu.2025.1626548

**Published:** 2025-05-30

**Authors:** Jiannan Zheng, Mingjun Du, Wei Ye, Jiaheng Xie, Pengpeng Zhang, Chenjun Huang, Haoran Lin

**Affiliations:** ^1^ Department of Thoracic Surgery, The First Affiliated Hospital with Nanjing Medical University, Nanjing, China; ^2^ Department of Plastic Surgery, Xiangya Hospital, Central South University, Changsha, China

**Keywords:** inflammation, single-cell sequencing, molecular biomarkers, immune microenvironment, precision medicine

Inflammation is a fundamental physiological response of the body to injury and infection, orchestrated by dynamic regulation across multilayered genetic, epigenetic, and cellular signaling networks to restore internal homeostasis. Acute inflammation helps to defend against pathogen invasion and repair tissue damage. However, if the inflammatory response fails to resolve properly or becomes dysregulated, it may develop into chronic inflammation. Persistent or aberrantly activated inflammation has been closely associated with the pathogenesis and progression of numerous major diseases, including cardiovascular diseases, autoimmune diseases, neurodegenerative disorders, cancer, and sepsis (1, 2). In-depth exploration of the molecular mechanisms of inflammation, cellular heterogeneity, and their regulatory networks has thus become a forefront of life sciences and medical research.

The inflammatory microenvironment encompasses various immune and non-immune cells, including neutrophils, macrophages, T cells, endothelial cells, and fibroblasts. These cells interact through chemokines, cytokines, and diverse receptor signaling pathways, working together to initiate, amplify, and resolve inflammation (3). During the acute phase, neutrophils directly engage in phagocytosis and elimination of pathogens, while macrophages subsequently mediate the clearance and repair of the microenvironment. In the chronic phase, the accumulation of regulatory T cells (Tregs), myeloid-derived suppressor cells (MDSCs), and other immunosuppressive cells can prevent excessive inflammatory reactions but may also result in immune dysfunction, tissue injury, and even fibrosis (4). Molecular biomarkers related to inflammation have become increasingly important in disease classification, prognosis monitoring, and the field of precision medicine. In recent years, emerging technologies that integrate single-cell sequencing and multi-omics analyses have enabled researchers to dissect the inflammatory microenvironment from multiple dimensions, including cellular subpopulations, gene expression, and spatial localization (5, 6).

New strategies targeting the inflammatory response are continually being developed, including pro-resolving mediators, cytokine inhibitors, immune cell therapies, and combination multi-target approaches (7). Several key studies in this Research Topic demonstrate significant advances in this field. Feng et al. explored the protective role of muscone in the development of chronic obstructive pulmonary disease (COPD) by establishing a mouse COPD model, finding that muscone significantly improved lung function, upregulated anti-inflammatory cytokines including IL-38, while inhibiting pro-inflammatory factors such as CXCR3, IFN-γ, IL-17A, and RORγt, providing a new therapeutic perspective for inflammatory regulation in COPD. Chen et al. investigated the effects of autologous platelet-rich plasma (PRP) treatment in patients with chronic endometritis (CE), demonstrating that PRP significantly reconstructed the uterine local immune microenvironment by reducing the proportions of CD8+ T cells, CD56+ NK cells, Foxp3+ Treg cells, and T-bet+ Th1 cells, while upregulating endometrial receptivity-related genes, thereby improving pregnancy outcomes (8). Guo et al., through a review of skin-homing T cells in recurrent episodes of atopic dermatitis, elucidated the key mechanisms of lymphocyte skin homing in disease recurrence, providing new intervention targets for chronic inflammatory skin diseases. With advances in single-cell sequencing and transcriptome analysis, Liu et al. revealed the mechanistic roles of sialylation-related genes CD19 and GPR65 in sepsis-induced acute respiratory distress syndrome, identifying CD14 monocytes as the key cell type (9), while Yang et al. comprehensively analyzed diagnostic biomarkers and immune cell infiltration features in sepsis through machine learning and bioinformatics techniques, identifying CD40LG as a key gene and target of the Chinese medicine Xuebijing. Additionally, Hao et al. investigated the role of mitophagy-related genes in acute myocardial infarction and ischemic cardiomyopathy, finding significant differences in the TGFβ pathway between high and low-risk groups, and validating RPS11 as an important diagnostic biomarker. Collectively, these studies advance the development of early warning, stratified diagnosis, and personalized treatment strategies for inflammation-related diseases, providing a solid foundation for clinical translation and disease prevention.

Research on the molecular mechanisms and therapeutic strategies of inflammation stands at a pivotal stage of rapid transformation. The application of single-cell and multi-omics technologies continues to fuel innovation in understanding disease mechanisms, diagnosis, and intervention strategies. The collection of cutting-edge studies presented in this Research Topic demonstrates significant advances in our understanding of inflammatory processes across different disease contexts. We anticipate that these findings will promote deep integration between basic and clinical research in inflammatory diseases, foster the development of innovative therapeutic approaches, and ultimately achieve precision treatment, benefitting a broader patient population.
